# What If Healthy Aging Is the ‘New Normal’?

**DOI:** 10.3390/ijerph14111389

**Published:** 2017-11-15

**Authors:** Marcia G. Ory, Matthew Lee Smith

**Affiliations:** 1 Center for Population Health and Aging, Texas A&M Health Science Center, College Station, TX 77843, USA; matthew.smith@tamhsc.edu; 2 College of Public Health, The University of Georgia, Athens, GA 30602, USA

## 1. Tribute

We dedicate this special issue to our colleague, Dr. Lucinda Bryant (1941–2016). She was a founding member of the United States Centers for Disease Control and Prevention Healthy Aging Research Network (CDC HAN), as well as a dear friend, colleague, mentor, and inspiration to so many. She dedicated her professional life to examining how older people defined their health and, in turn, helped redefine what it means to grow older with grace and wisdom, and strategies for doing so.

## 2. Editorial

Global aging is a relatively new phenomenon, but one that is catching the attention of researchers, practitioners, and policy makers around the world [1]. As fertility and mortality rates are declining in most countries, the rapid aging of populations worldwide is resulting in unprecedented changes for individuals, their families, and governments. The ‘new normal’ will be a world with more persons aged 65 and older than children under age five, with most dramatic age-related disproportions occurring in developing countries [2]. The sheer numbers are ‘game changing’ with the older population projected to grow from an estimated 524 million in 2010 to nearly 1.5 billion in 2050 [2]. Thus, there is little doubt that there will be more older people on earth who, on average, are living longer than earlier generations. Among the biggest issue will be how societies can foster healthy aging [3], and whether we can translate research successes into practice so that healthy aging will become the new normal in a rapidly aging world [4].

For this special issue, we solicited papers about aging and health promotion to further a global understanding of the causes of, and interventions for, healthy aging. This special issue includes a wide range of research-based articles with a focus on the interrelationships among aging, health, and environmental factors. The articles reflect diverse methodologies reflecting the value of different approaches for addressing key questions about the determinants and consequences of different risk factors over the life-course. Furthermore, some of the articles examine the impact of different intervention strategies for enhancing the lives of older adults around the world. For example, there are observational and intervention studies; longitudinal and cross-sectional studies; quantitative and qualitative studies; laboratory and field studies; consideration of both individual and environmental determinants; subjective and objective measurements (ranging from environmental assessments to neuroimaging); and implications for a host of multi-level intervention strategies.

This collection of articles confirms several basic principles about aging research and practice. We see the heterogeneity of the older population, with clear insights into differences in aging by individual and social factors. Longitudinal research studies are especially valuable for documenting life-course trajectories, revealing the dynamic nature of interacting health and psychosocial processes, and observing the continued potency of risk factors (e.g., being poor or lacking social ties) on functional health and well-being [5]. Additionally, social engagement seems to be important for later-life cognitive function trajectories, resulting in heterogeneous trajectories that also mediate the combined impact of cumulative life exposures [6]. Social support is a complex concept, thus highlighting the importance of understanding the impacts of different support types. Other longitudinal research shows the impact of multiple chronic conditions on depression for older adults may be differentially impacted by positive and negative social support [7]. Furthermore, social capital has been associated with performance of activities of daily living and mortality, although these relationships differ by participation at the individual versus community level and by gender [8].

Expanding previous aging research that typically focuses on individual health determinants, several articles take a socio-ecological approach focused on the role of different individual and physical environmental factors on residential, health, and behavioral outcomes. “Aging-in-Place” is preferred by the majority of adults around the world. In the US, use of home- and community-based services are often necessary to help older adults “age-in-place”, which suggests the need for expanded services to address older adult’s physical and mental health demands [9]. The environment can be viewed as a multi-dimensional concept including family, home, finances, neighborhood, and health care. These environmental factors interact with individual factors to influence the physical and mental health of older adults [10]. Furthermore, environmental stresses such as oxidative stress and heat stress are associated with increased mortality in laboratory studies signaling potential aging mechanisms in humans [11].

Physical activity is seen as one of the strongest predictors of health and functioning in later life, with the emerging recognition that “Exercise is Medicine”. Thus, it is not surprising that several articles examine environmental influences on different levels of physical activity, and findings across several countries have similar themes. The determinants and consequences of sedentary behavior are gaining recognition as an independent risk factor. In addition, neighborhood environmental factors associated with sedentary behavior among retirees using objective measures can help guide interventions [12]. Methodological advances in conducting street audits are helpful to identify the impact of different microscale environmental features on walking patterns among older adults [13,14].

Other studies are examining the confluence of physical and social environmental factors. The relationship, for example, between park proximity and recreational physical activity is mediated social trust and cohesion [15]. Inequalities in neighborhood safety, pedestrian infrastructure, and aesthetics are seen to impact walking (i.e., especially older adult’s outdoor walking) and call for improvements in high-deprivation areas [16]. Neighborhood factors are also seen to increase the risk of fall-related injuries, which emphasizes the protective effects of stable and safe neighborhoods, despite these effects not varying by individual characteristics such as age [17]. Additionally, neighborhood social cohesion has been shown to decrease fall risk among community-dwelling older adults, independent of demographic and health-related covariates [18]. Especially innovative environmental psychology research uses a mixed-methods approach to examine the impact of walking on brain activity in different types of urban environments [19].

We were especially interested in papers that would advance our knowledge about the needs of and strategies for promoting health among underserved older populations. Such attention is important because these populations are often overlooked in research studies or not typically reached by health promotion programs. For example, although rural areas are disproportionately older, programmatic efforts often do not reach those in rural areas; therefore, we know little about intervention delivery characteristics in rural or non-metropolitan areas [20]. Examining factors associated with those foregoing medical care due to cost barriers is important for improved intervention targeting and can help pinpoint strategies to bridge the gaps in access to care [21]. Similarly, identifying individual- and place-based characteristics associated with access to care for specific medical diagnoses in diverse populations can help identify risk groups (e.g., racial/ethnic adults, economically disadvantaged) and places (e.g., the South, rural areas) for the delivery of chronic disease self-management programs [22]. Meeting non-emergency medical transportation needs is a challenge for many older adults who no longer drive, but especially challenging for those living in rural areas who need essential medical services such as dialysis [23].

This special issue further confirms the universality of aging issues, and the similarities and differences across different countries in addressing the forthcoming ‘grey tsunami’ with articles reflecting about aging in the United States, the United Kingdom, Europe, Australia, and Asia. The one-child policy in China raises interesting questions about the existence of social ties and impacts of the more prevalent ‘empty nest’ scenario with clear differences in self-reported health status across identifiable demographic characteristics such as age, sex, and rurality status [24]. The same study provides a more in-depth examination of objective clinical outcomes in one geographic setting, suggesting fewer impacts on actual biological markers [25].

Most importantly, this special issue serves to debunk the stereotypic view that older adults are not interested in, or cannot benefit from, health promotion interventions. Contrary to popular belief, health promotion is not anathema to aging; it should occur throughout the life-course. It is never too late to adopt healthy behaviors or discard unhealthy ones, become more socially engaged, or reap benefits from more supportive environments.

The next generation of ‘exergame’ interventions that combine computer gaming systems with an interface that requires physical exertion to play is showing how a person-centered co-learning approach can have many positive health benefits [26]. Robotics are proving to be a promising technological solution for motor and cognitive rehabilitation [27]. While there are many community-based evidence-based programs for older adults, less is known about the cost-effectiveness of these programs relative to other intervention strategies. Evidence is emerging for the cost-effectiveness of lifestyle programs that are designed to improve physical activity and dietary behaviors [28].

This collection of work is important in that it captures and integrates excellent research that provides a better understanding of global aging and the dynamic role of interacting health, aging, and environmental factors. As guest editors for this special issue and Directors of the newly established Center for Population Health and Aging (https://cpha.tamhsc.edu/) within the Texas A&M Health Science Center, we applaud the individual authors on their contributions to advance science and health promotion among older adults. Together, we look forward to proactive personal, familial, and societal actions to encourage healthy aging so that it will indeed become the ‘new normal’.
